# Serum cytokine profiling reveals CXCL10 (IP-10) as a major predictor of severe COVID-19 outcomes in hospitalized patients during the first pandemic wave in Italy

**DOI:** 10.3389/fimmu.2026.1816573

**Published:** 2026-05-25

**Authors:** Anita Muglia, Daniele Petrone, Ilaria Schiavoni, Letizia Santinelli, Pasqualina Leone, Antonino Bella, Gabriella d’Ettorre, Francesco Alessandri, Anna Teresa Palamara, Claudio Maria Mastroianni, Paola Stefanelli, Giorgio Fedele

**Affiliations:** 1 National PhD Programme in One Health approaches to infectious diseases and life science research, Department of Public Health, Experimental and Forensic Medicine, University of Pavia, Pavia, Italy; 2 Department of Infectious Diseases, Istituto Superiore di Sanità, Rome, Italy; 3 Department of Public Health and Infectious Diseases, Sapienza University of Rome, Rome, Italy; 4 Department of Anaesthesia and Intensive Care, Sapienza University of Rome, Rome, Italy

**Keywords:** biomarkers, COVID-19, CXCL10/IP-10, disease severity, innate immunity

## Abstract

**Introduction:**

Coronavirus disease 2019 (COVID-19) severity is closely associated with dysregulated inflammatory responses, with cytokines and chemokines emerging as key mediators and potential early biomarkers of adverse clinical outcomes. A retrospective study on 103 RT-PCR-confirmed COVID-19 patients during the first pandemic wave (January-May 2020) was performed to evaluate the prognostic value of a panel of cytokines and chemokines measured at hospital admission.

**Methods:**

Samples and clinical data were collected at the Department of Public Health and Infectious Diseases, Sapienza University of Rome, and sent to Istituto Superiore di Sanità for further characterization. Serum concentrations of IL-1β, IL-6, IL-8, IL-10, TNF-α, CCL3, and CXCL10 (IP-10) were quantified using multiplex ELISA. Associations with in-hospital mortality, intensive care unit (ICU) admission, and a composite outcome (death or ICU admission) were assessed using multivariable logistic regression.

**Results:**

ICU admission occurred in 6.8% of patients and mortality in 11.7%. Among inflammatory mediators, CXCL10 emerged as the strongest predictor of adverse outcomes. In adjusted models, each 1,000 pg/mL increase in CXCL10 was associated with increased odds of death (OR 1.26; 95% CI 1.17-1.35), ICU admission (OR 1.13; 95% CI 1.06-1.21), and the composite outcome (OR 1.21; 95% CI 1.12-1.31). Elevated respiratory frequency and blood urea nitrogen were also independently associated with worse outcomes, while TNF-α tended to be associated with ICU admission. Conversely, IL-6 and other cytokines were not significant predictors in the multivariable models.

**Discussion:**

These findings identify CXCL10 as a key early immunological predictor of COVID-19 severity, suggesting that its integration with clinical parameters may improve risk stratification and guide targeted management. CXCL10 may also represent a potential therapeutic target, warranting validation in larger prospective studies.

## Introduction

Coronavirus disease 2019 (COVID-19) pandemic has been one of the greatest public health challenges in recent human history. COVID-19 is a highly infectious disease caused by severe acute respiratory syndrome coronavirus 2 (SARS-CoV-2) which, since its emergence to October 2025, has resulted in approximately 7.1 million deaths worldwide (1). The clinical manifestations associated with this disease, are extremely heterogeneous, ranging from asymptomatic cases to serious illness. In the most severe cases, patients may suffer from acute respiratory distress syndrome (ARDS), pneumonia and even multiple organ failure which may be related to an unbalanced immune response (2–4). The boundary between the various stages of disease severity is blurred and largely contingent on factors such as age, presence of comorbidities and host factors, as well as the infecting variant (5). During an acute respiratory viral infection, the host’s resistance mounts a complex protective immune response characterized by a crosstalk between supportive and detrimental inflammatory and antiviral mechanisms. In COVID-19, progression to severe disease is closely associated with dysregulated immune activation (6). Patients with severe disease may exhibit a phenomenon known as a “cytokine storm”, characterized by excessive production of pro-inflammatory mediators (7). The excessive and uncontrolled production of pro-inflammatory mediators leads to widespread pulmonary oedema, alveolar damage, and the development of acute respiratory distress syndrome (ARDS). In addition, inflammation promotes activation of the coagulation cascade, resulting in microthrombi formation in pulmonary capillaries and impaired oxygen exchange (3–7).

A pattern of biomarkers, commonly used in clinical practice, has been identified in patients with severe COVID-19 compared to mild systemic disease and this justify their inclusion in risk stratification models. Among these, hematologic biomarkers include neutrophil-to-lymphocyte ratio (NLR), serum C-reactive protein (CRP), D-dimer, lymphocyte count, platelet count, white blood cell (WBC) count and hemoglobin (8–13). The NLR, calculated simply by the ratio of neutrophils count/lymphocytes count, is an inflammatory marker that, for COVID-19 patients, has been shown to be an independent risk factor for severe disease (14). Patients with high on-admission serum CRP levels are more likely to develop severe conditions (15); specific cut-off values for both NLR and platelet-to-lymphocyte ratio (PLR) have been shown to predict mortality, the need for ventilation and admission to intensive care (ICU), and their trend correlates with disease severity (16). The early identification of serum biomarkers is critical to promptly initiate targeted therapies and tools are needed for early risk assessment (17). Despite the essential role of cytokines and chemokines in driving the systemic inflammation, observed in severe COVID-19, their measurement is often reserved for research settings due to the need for specialized, resource-intensive assays and the lack of standardized methodologies for reliable clinical implementation (18). However, since the beginning of the pandemic, several studies attempted to investigate the role of these inflammatory mediators as predictors of disease outcome. Evidence indicates that clinical outcomes are closely linked to the magnitude, kinetics, and balance of the immune response. In fact, circulating inflammatory cytokines and chemokines, such as IL-6, CCL2, CCL3, CXCL10, IL-10, IL-23, TNF-α, IL-18, M-CSF, and MCP-3 have been associated with increasing disease severity and mortality (19–26). Furthermore, these mediators appear to play a key role during SARS-CoV-2 infection, not only in the lung pathology, but also in systemic vascular damage and endothelial dysfunction (27) A recent *in vitro* study shows that CXCL10 plays a crucial role in COVID-19-related vasculopathy, driving an epithelial-endothelial crosstalk, which leads to endothelial activation, increased vascular permeability and recruitment of inflammatory cells (28). This effect was also observed in absence of direct infection, suggesting that vascular damage is largely driven by the host inflammatory response rather than direct viral cytopathic effect (28). Although the role of cytokines and chemokines in the development of severe disease in SARS-CoV-2-infected patients is well established, it remains challenging to identify a reliable multivariate panel of inflammatory biomarkers, measured in the early stages of the disease, capable of independently predicting an unfavorable prognosis.

To address this gap, this study aimed to provide a comprehensive analysis comparing plasma levels of a panel of seven cytokines and chemokines in relation to disease severity and clinical outcome. We also sought to identify the individual inflammatory mediators with the highest predictive potential for mortality and poor prognosis using a multivariate statistic model.

## Materials and methods

### Study design and population under study

**Figure 1:**
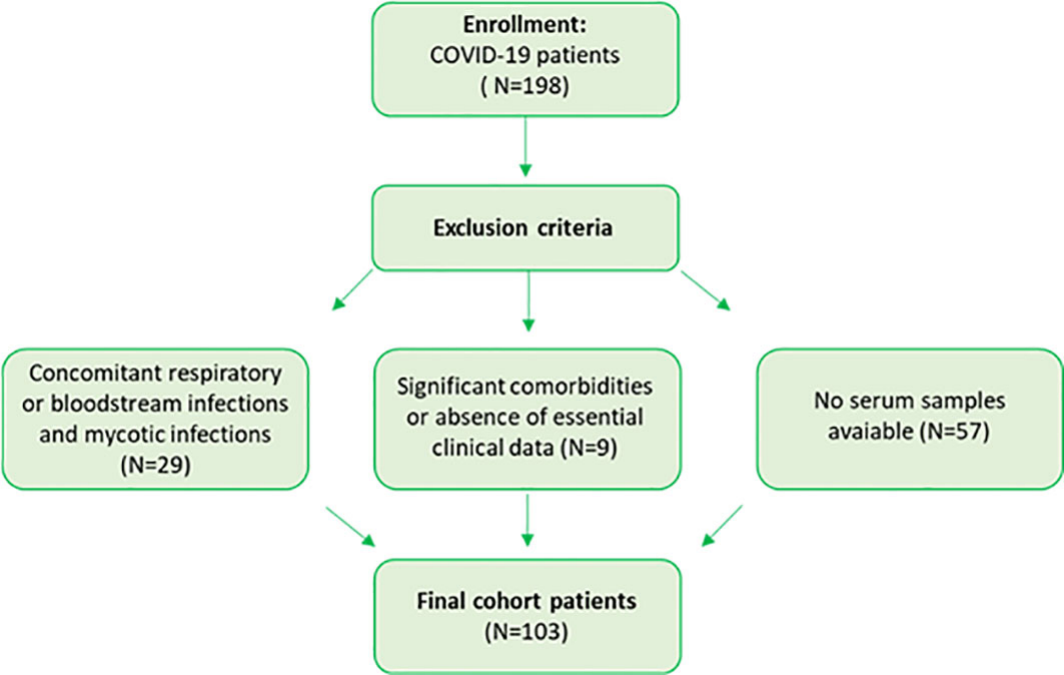
Study flow diagram. This flow diagram illustrates the selection process for the study cohort. A total of 198 patients with confirmed cases of SARS-CoV-2 were initially assessed for eligibility. Patients were excluded if they had concomitant respiratory, bloodstream or fungal infections (n = 29), significant comorbidities or missing essential clinical data (n = 9), or if serum samples were unavailable for biomarker analysis (n = 57). The final study population consisted of the 103 patients who were included in the statistical analyses.

We retrospectively analyzed the clinical database of 198 patients with confirmed COVID-19, defined by a positive real-time reverse transcription polymerase chain reaction (RT-PCR) test (RealStar SARS-CoV-2 RT-PCR; Altona Diagnostics, Hamburg, Germany), who were admitted to Umberto I Hospital at Department of Public Health and Infectious Diseases, Sapienza University in Rome between January and May 2020, during the first wave of the pandemic. The study was approved by the Institutional Ethics Committee of CET Lazio Area 1, Rome (Rif. 5965, Prot. 0750/2020), and written informed consent was obtained from all participants prior to their inclusion in the study. Among these patients, 29 were excluded from the study cohort due to documented concomitant respiratory or circulatory infections caused by other pathogens, including fungal infections; 9 were excluded because of significant comorbidities that could have independently led to hospitalization or confounded the analysis, or because essential clinical data were missing; and 57 were excluded because serum samples were no longer available. Consequently, the final cohort comprised 103 patients (**Figure 1**).

### Clinical and laboratory data collection

Clinical data were retrieved from medical records at hospital admission, including demographic and clinical characteristics, comorbidities, laboratory test results, biochemical, and immunological parameters, composite clinical severity scores and treatments administered.

### Clinical severity scoring

To assess baseline disease severity and comorbidity burden, we calculated established clinical scores for each patient at hospital admission. The Charlson Comorbidity Index (CCI) (29) was used to quantify pre-existing comorbidities and estimate 10-year mortality risk. Pneumonia severity was evaluated using the Pneumonia Severity Index (PSI) (30) and the standard CURB-65 scores (31). Additionally, an expanded CURB-65 score (32), incorporating additional clinical and laboratory parameters, was computed allowing for a comparative analysis between traditional scoring systems and more comprehensive risk-stratification tools.

### Study outcomes

We investigated the association between severe COVID-19 outcome (death, ICU admission, and a composite of the two) and a range of patient characteristics, including demographic, clinical, laboratory, and seven cytokines measured in serum at hospital admission.

### Serum preparation, storage, and cytokine/chemokine quantification

Serum samples were obtained by collecting peripheral blood (5 mL) in Serum Separator Tubes (BD Diagnostic Systems, Franklin Lakes, NJ, USA); they were then centrifuged at 3000 rpm for 10 min at room temperature. Two aliquots of purified serum for each donor were transferred to 2mL polypropylene, screw-cap cryo tubes (Nunc™, Thermofisher Scientific, Waltham, MA USA). Aliquots were immediately frozen at -20 °C and subsequently stored at -80°C. Frozen serum samples were shipped on dry ice to the Department of Infectious Diseases at the Istituto Superiore di Sanità (DMI, ISS), following biosafety shipment conditions. Upon arrival, serum samples were immediately stored at -80 °C. Before analysis, serum samples were heat-inactivated at 56 °C for 30 minutes and diluted 1:2 using the assay diluent provided within the Ella system cartridges. Quantitative determination of cytokines and chemokines was performed using a customized next-generation multiplex ELISA on the Ella Automated Immunoassay System (Bio-Techne, Minneapolis, MN), according to the manufacturer’s instructions. The assay cartridges were customized to simultaneously detect IL-1β, CXCL10, CCL3, TNF-α, IL-6, IL-8, and IL-10 (internal assay identifier, SPCKE-PS-014549 V5). A standard curve was generated for each cytokine using pre-loaded calibration standards within the system cartridges. Positive and negative controls were included as part of the system to ensure the reliability and precision of the assay, according to the manufacturer’s instructions. The assay was validated for use with human serum, ensuring consistent and reproducible measurements across samples.

### Statistical analysis

We described the main characteristics of cases and the frequency of the outcomes of interest using counts and percentages. Continuous variables were summarized as mean and standard deviation (SD) or median and interquartile range (IQR), while categorical variables were reported as absolute and relative frequencies. **Supplementary Table S1** reports the list of the variables collected and included in the analysis, together with their description. Additional variables were derived as follows: number of comorbidities (from 0 to 6), presence of comorbidities (Yes/No), age group in years (20-49, 50-64, 65-74, 75-84, 85+), albumin 35+ g/L (Yes/No), NLR (obtained as neutrophil/lymphocyte ratio), PLR (as platelet/lymphocyte ratio) and DPR (as D-dimer/platelet ratio).

Then, we categorized each cytokine in 5 groups, according to their individual empirical distribution. Tertile-based thresholds, as well as minimum and maximum values, were calculated using non-zero observations only. To summarize the inflammatory burden, cytokines concentrations were categorized into five ordinal classes (0–4) which were used to build an inflammatory score (33, 34). To the values equal to zero were assigned a score of 0, while other values were assigned scores ranging from 1 to 4 according to the distribution. Due to the anti-inflammatory role of IL-10, two scores were obtained: one obtained by summing the scores of the six pro-inflammatory cytokines (score 1), and a second score derived by subtracting the IL-10 score from this sum (score 2). Based on preliminary findings, the role of CXCL10 was further explored by defining two additional scores analogous to the previous ones but excluding this cytokine (score 3 – score 4).

To facilitate clinical interpretability, the four composite scores were analyzed both as continuous variables and as categorical variables. Categorical versions were obtained using tertile-based cut-offs (low, intermediate, and high inflammatory burden) and median-based dichotomization. All score formulations were carried forward to the analysis.

We estimated the association between the cytokines and the three outcomes (death, admission in ICU, composite outcome) using separate multivariable logistic regression model for each outcome. As a first step, univariate logistic regression analyses were performed for each outcome to evaluate the association between individual covariates and the outcome of interest. Based on these results, candidate variables for multivariable models were identified. Covariates showing an association, i.e. odds ratio (OR), with a p-value less than or equal to 0.20 in univariate analyses were considered eligible for inclusion in the corresponding multivariable models. Multivariable logistic regression models were then constructed separately for each outcome; the final choice of variables included was performed using a backward selection procedure guided by the Akaike Information Criterion (AIC). Estimates from the logistic models are presented with their 95% confidence interval (95% CI). For variables with a percentage of missing values ​​not exceeding 15%, multiple imputation was performed via predictive mean matching (PMM), generating 5 imputed datasets. Final estimates were combined using Rubin’s rules to account for imputation uncertainty (35).

For ease of interpretation and readability of the results, selected continuous variables were rescaled prior to analysis. Specifically, respiratory frequency (RF) and blood urea nitrogen (BUN) were divided by 10 to express Ors per 10-unit increase; TNF-α serum levels were divided by 100; WBC and CXCL10 concentrations were divided by 1,000. Accordingly, all reported Ors refer to these rescaled units.

All the analyses were carried out with Rstudio 2024.04.2 under R 4.4.1.

## Results

### Clinical and demographic characteristics

The final study cohort of 103 patients with confirmed COVID-19 comprised 43 women (41.8%) and 60 men (58.2%). The median age was 62 years (IQR: 55-71), with no significant difference between sexes (p = 0.30).

The CCI reflected these significant yet heterogeneous pre-existing conditions, with most patients scoring 1 (27.2%) or 2 (22.3%). Assessment of clinical severity upon admission showed a mean PSI score of 70.7 (SD ± 26.5). Risk stratification using the standard CURB-65 scale indicated predominantly mild-to-moderate severity, with all evaluable patients scoring between 0 and 2. In contrast, the Expanded CURB-65 score system highlighted a greater degree of biological impairment and 36.9% of patients were reclassified into severe risk categories (score ≥ 3). The median length of hospital stay was 15 days (IQR: 11-27) (**Table 1**). Additional characteristics of the study population, including treatment variables and other collected measures not detailed here, are reported in **Supplementary Table S2**.

**Table 1:** Distribution of the demographic and clinical characteristics of the study population.

Characteristics		Cases N (%)
Gender	Male	60 (58.25%)
	Female	43 (41.75%)
Age, Years (Median [IQR])		62 [55 - 71]
Age, Years	≤65 Years	60 (58.25%)
	>65 Years	43 (41.75%)
RF (Median [IQR])		18 [16 - 20]
Hypoxemia	Yes	3 (2.91%)
	No	100 (97.09%)
Charlson index	0	15 (14.56%)
	1	28 (27.18%)
	2	23 (22.33%)
	3	8 (7.77%)
	4	14 (13.59%)
	5	6 (5.83%)
	6	4 (3.88%)
	7	2 (1.94%)
	8	1 (0.97%)
History of autoimmune disease	Yes	2 (1.94%)
	No	94 (91.26%)
CURB-65 score	0	31 (30.10%)
	1	34 (33.01%)
	2	34 (33.01%)
Extended CURB-65 score	0	13 (12.62%)
	1	23 (22.33%)
	2	25 (24.27%)
	3	24 (23.3%)
	4	13 (12.62%)
	5	1 (0.97%)
PSI (Median [IQR])		68 [53 - 85]
Hospital length of stay (days) (Median [IQR])		15 [11 - 27]
Oxygen therapy	Yes	55 (53.40%)
	No	34 (33.01%)
CPAP ventilation	Yes	10 (9.71%)
	No	79 (76.70%)

Data in parentheses represent percentages or Inter-Quartile Range (IQR). The rates are presented to two decimal places and are calculated on 103 units, the number of patients included in the population study. RF, Respiratory Frequency; Hypoxemia, PaO_2_ < 60 mmHg and SpO_2_ < 90%, PSI, Pneumonia Severity Index; CPAP ventilation, Continuous Positive Airway Pressure (CPAP) ventilation.

At hospital admission, more than half of the study population (61.7%) presented with one or more comorbidities, including cardiovascular (37.9%), metabolic (20.4%), neoplastic (12.6%), respiratory (10.7%), neurological (6.8%) and renal (3.9%) diseases. During the observation period, 7 out of 103 patients (6.8%) were admitted to the intensive care unit (ICU), and 12 patients (11.7%) died. Notably, a strong association was observed between disease severity and mortality: 5 of 7 ICU patients (71.4%) died. Conversely, the remaining 7 deaths occurred without prior ICU admission. The comorbidities and clinical outcomes of all participants are shown in **Table 2**.

**Table 2:** Distribution of comorbidities and outcomes of the study population.

Characteristics		Cases N (%)
Neoplastic	Yes	13 (12.62%)
	No	90 (87.38%)
Cardiovascular	Yes	39 (37.86%)
	No	64 (62.14%)
Respiratory	Yes	11 (10.68%)
	No	92 (89.32%)
Renal	Yes	4 (3.88%)
	No	99 (96.12%)
Metabolic	Yes	21 (20.39%)
	No	82 (79.61%)
Neurological	Yes	7 (6.80%)
	No	96 (93.20%)
Presence of comorbidities	Yes	63 (61.17%)
	No	40 (38.83%)
Number of comorbidities (Median [IQR])		1 [0 - 1]
ICU admission	Yes	7 (6.80%)
	No	96 (93.2%)
Death	Yes	12 (11.65%)
	No	91 (88.35%)
Composite outcome	Yes	14 (13.59%)
	No	89 (86.41%)

Data in parentheses represent percentages or inter-quartile range (IQR). The rates are presented to two decimal places and are calculated on 103 units, the number of patients included in the population study.

### Biochemical and hematological characteristics upon admission

At hospital admission, patients’ biochemical and hematological profiles showed that the total WBC count was within the normal range (median [IQR]: 5,440/µL [4.325-6.975]), but a marked lymphopenia was observed, with a median lymphocyte count of 1,047 cells/µL (± 667) and a median value of 850 cells/µL Increased levels of established markers of systemic inflammation and tissue damage, including C-reactive protein (CRP), D-dimer, and lactate dehydrogenase (LDH), supported the biological impairment suggested by clinical severity scores. In particular, 47.6% of patients had CRP levels > 41.8 mg/L. D-dimer levels (median [IQR]: 1,081 ng/mL [502-1,686]), assessed as an indicator of coagulation activation, were elevated, while platelet counts (median [IQR]: 201,000/µL [162,500-243,500]), reflecting hemostatic status, remained within the normal range. LDH values (median [IQR]:257 U/L [228-392]), measured as a marker of tissue damage, were also increased. (**Table 3**).

**Table 3:** Distribution of the biochemical and hematological characteristics upon admission of the study population.

Characteristics		(Median [IQR])
WBC		5,440 [4,325 - 6,975]
Neutrophil count		3,930 [2,785 - 5,126]
Lymphocyte count		850 [645 - 1,205]
Monocyte count		310 [240 - 415]
CRP > 41.8 mg/L	Yes (N %)	49 (47.57%)
	No (N %)	54 (52.43%)
D-dimer		1,081 [502 - 1,686]
Albumin		38 [34 - 42]
LDH		257 [228 - 392]
Platelet count		201,000 [162,500 - 243,500]
BUN		9 [6 - 14]
Glucose		81 [6 - 104]
Sodium		138 [136 - 140]

Data in parentheses represent percentages or Inter-Quartile Range (IQR). The rates are presented to two decimal places and are calculated on 103 units, the number of patients included in the population study. WBC, White blood cell count; LDH, Lactate Dehydrogenase; BUN, Blood Urea Nitrogen.

### Cytokine and chemokine profile of COVID-19 patients

**Figure 2:**
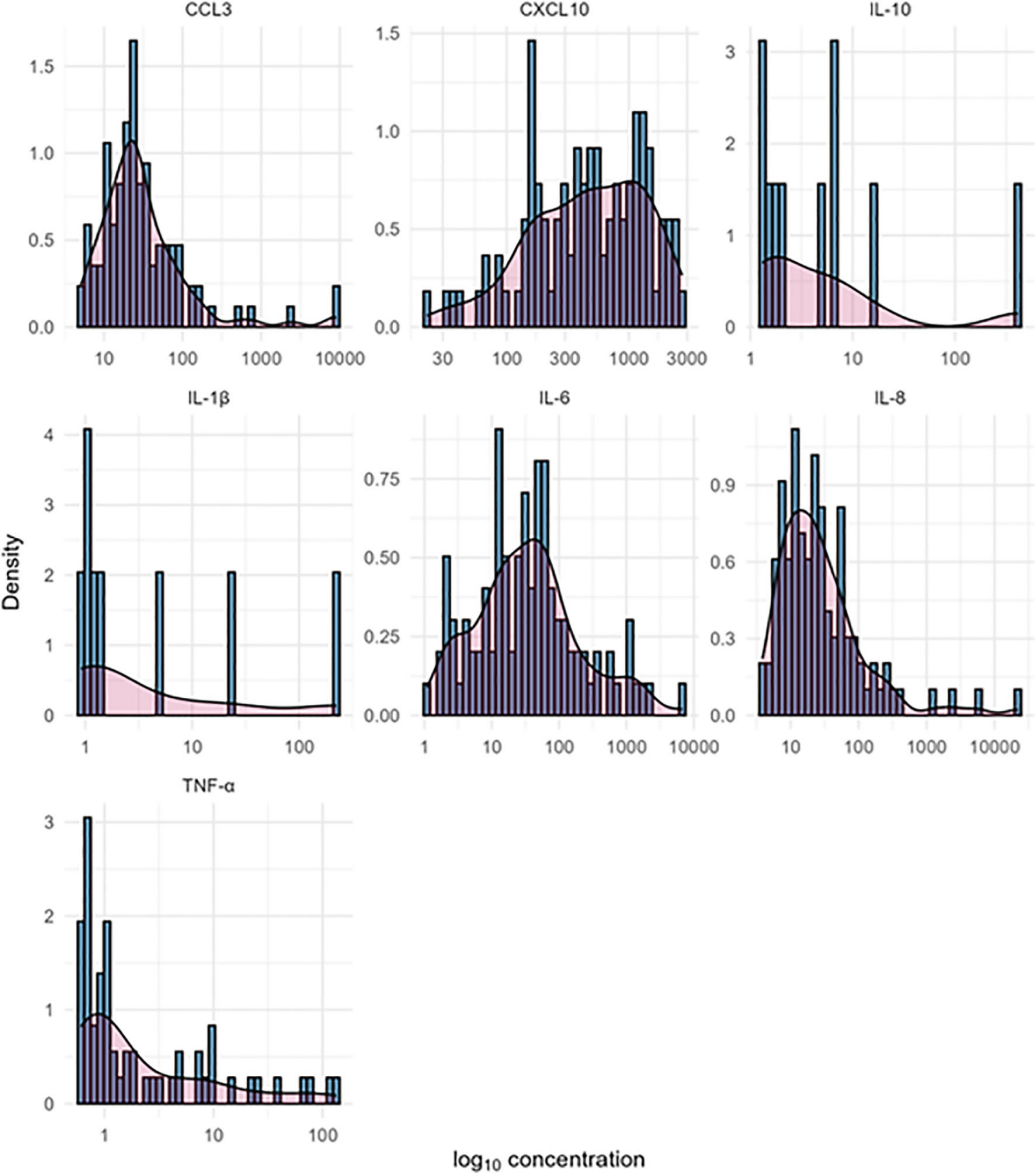
Histograms and density plots of cytokine concentrations on a logarithmic scale. The histograms show the distribution of seven circulating inflammatory biomarkers (CCL3, CXCL10, IL-10, IL-1β, IL-6, IL-8 and TNF- α), as measured in serum samples from the study cohort (n = 103 for all except IL-6 and TNF-α, both of which have a missing value), with overlaid kernel density estimates. Cytokine concentrations were quantified using a customized next-generation multiplex ELISA and are expressed on a log10 scale to account for skewed distributions and wide dynamic ranges. The bars represent normalized frequencies (density), such that the total area under each histogram equals one, while the smooth curves indicate the kernel density estimates.

To characterize the patterns of the inflammatory response at hospital admission, we analyzed a panel of seven cytokines and chemokines in serum samples. The multiplex ELISA analysis revealed a pro-inflammatory profile dominated by elevated levels of CXCL10 (mean: 721.29 pg/mL ± 651.69) and IL-6 (mean: 216.93 pg/mL ± 744.14). IL-8 (mean: 347.67 pg/mL ± 2,244.79) and CCL3 (mean: 239.19 pg/mL ± 1,227.05) also showed high mean concentrations. Conversely, TNF-α levels (mean: 5.92 pg/mL ± 20.17) remained comparatively low and IL-1β (mean: 2.49 pg/mL ± 22.06) was almost never detectable. Notably, the anti-inflammatory cytokine IL-10 levels were also negligible (mean: 4.42 pg/mL ± 40.6) with values below the threshold limit in most patients (**Figure 2**) (**Table 4**). Finally, score 1 showed a mean value of 13.8 (± 3.77), and score 2 a mean value of 13.5 (± 3.56), both with a median of 13 (IQR: 5-22), highlighting marked clinical heterogeneity. The other two scores also showed similar results: score 3 had an average value of 10.83 (± 3.53) and score 4 had an average value of 10.54 (± 3.34), both with a median value of 10 (IQR: 8-13).

**Table 4:** Distribution of the cytokine and chemokine profile of the study population.

Characteristics	Median [IQR]
CXCL10	487 [182 - 1,118]
IL-6	30 [12 - 84]
IL-8	21 [10 - 51]
CCL3	23 [13 - 46]
TNF-α	1 [0 -1]
IL-1β	0 [0 - 0]
IL-10	0 [0 - 0]
SCORE 1	13 [11 - 16]
SCORE 2	13 [11 - 16]
SCORE 3	10 [8 - 13]
SCORE 4	10 [8 - 13]

Data in parentheses represent Inter-Quartile Range (IQR). The scores are based on the empirical distribution of the cytokines concentrations. They were categorized into five ordinal classes (0-4) which were used to build an inflammatory score. To the values equal to zero were assigned a score of 0, while other values were assigned scores ranging from 1 to 4 according to the distribution. Due to the anti-inflammatory role of IL-10, two scores were obtained: one obtained by summing the scores of the six pro-inflammatory cytokines (score 1), and a second score derived by subtracting the IL-10 score from this sum (score 2). Based on preliminary findings, the role of CXCL10 was further explored by defining two additional scores analogous to the previous ones but excluding this cytokine (score 3 - score 4).

### Univariate association analysis with death, ICU admission, and composite outcome

After the multiple imputation described above, the univariate statistical analysis showed that age >65 years was significantly associated with an increased risk of death (p = 0.021). A similar significant association was observed for the composite outcome (p = 0.006), whereas age was not significantly associated with the risk of ICU admission (p = 0.506). Elevated RF was associated with high odds ratios for all the three outcomes (p = 0.002 for ICU admission, p < 0.001 for the others).

Among biochemical markers, increased BUN (p = 0.006), glucose (p = 0.007), LDH (p = 0.012), D-dimer (p = 0.004), NLR (p = 0.034) and DPR (p = 0.004) together with a reduction in albumin levels (p = 0.005) emerged as significant predictors of death. Both WBC counts at diagnosis (p = 0.040), NLR (p = 0.023) and neutrophilia (p = 0.051) were significantly associated with ICU. For the composite severity outcome, markers of organ dysfunction such as BUN (p = 0.002) and glucose (p = 0.012), and indicators of systemic inflammation, such as high neutrophil counts (p = 0.030), NLR (p = 0.003), DPR (p = 0.007) and LDH (p = 0.025) were significant. Remarkably, among the seven cytokine and chemokine analyzed, CXCL10 emerged as the strongest immunological predictor of death (p < 0.001), ICU admission (p = 0.002), and composite outcome (p < 0.001). In contrast, other cytokines and chemokines analyzed did not show significant associations with any of the outcomes, nor the inflammatory scores, except TNF-α level (p = 0.183) with a potential association with ICU admission, highlighting the specific predictive value of CXCL10 in this cohort (**Supplementary Table S2**).

### Multivariable biomarkers of COVID-19 severity

**Figure 3:**
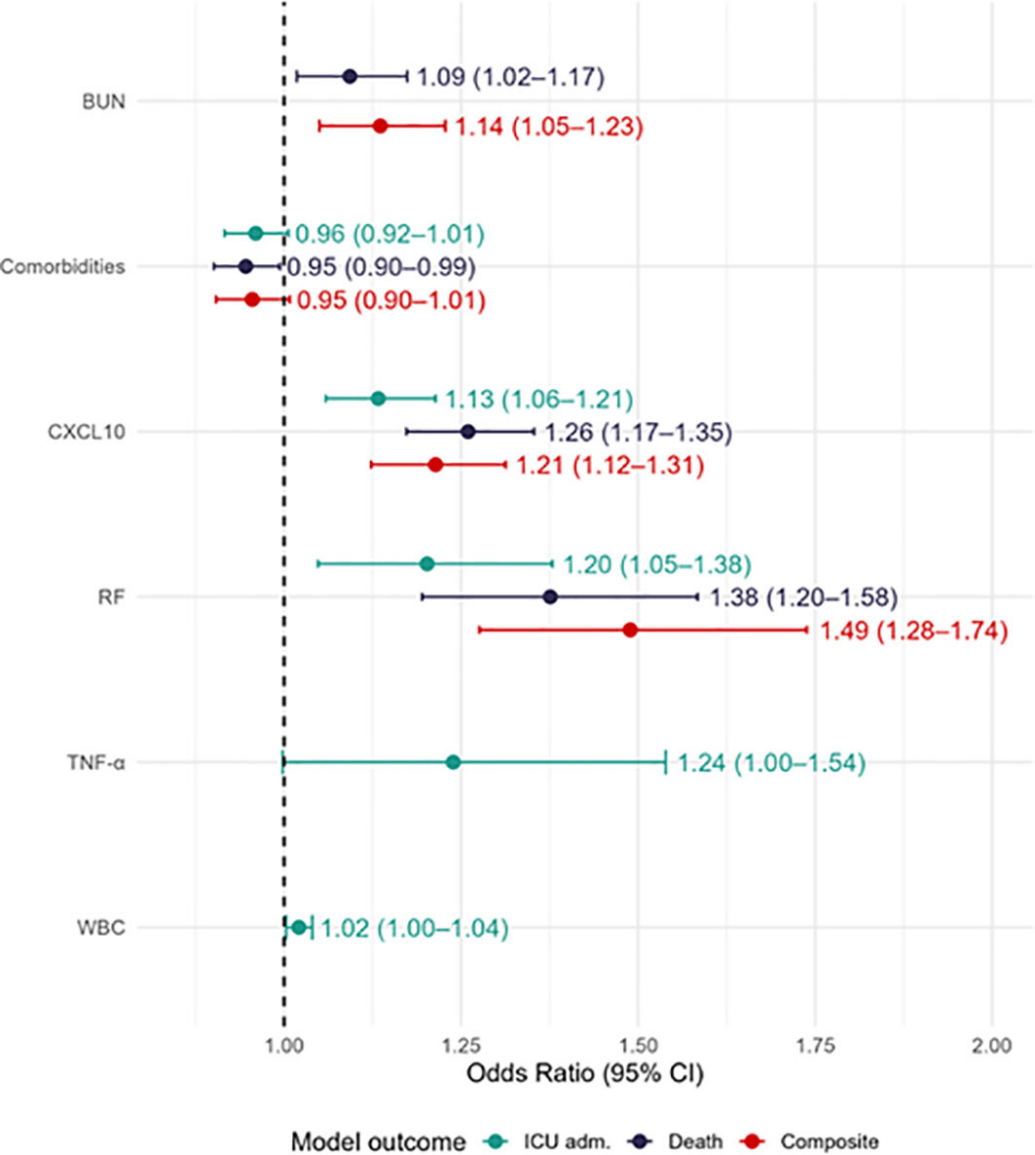
Adjusted odds ratios estimated with the logistic model for the study outcomes. Forest plot displaying adjusted odds ratios (ORs) with 95% confidence intervals (CIs) for the association between selected predictors and clinical outcomes, including intensive care unit (ICU) admission, death, and a composite endpoint, in the study cohort (n = 103). Biomarkers (CXCL10 and TNF-α) were measured in serum using a customized next-generation multiplex ELISA, while clinical and laboratory variables (Blood Urea Nitrogen [BUN], respiratory frequency [RF], white blood cell count [WBC], and number of comorbidities) were obtained from medical records at hospital admission. Point estimates are represented by dots, with horizontal lines indicating 95% CIs; the vertical dashed line denotes the null value (OR = 1). For variables with a percentage of missing values not exceeding 15%, multiple imputation was performed via predictive mean matching (PMM), generating 5 imputed datasets. Final estimates were combined using Rubin’s rules to account for imputation uncertainty. Predictor selection was performed separately for each outcome using a two-step approach: first, candidate variables were screened using univariable logistic regression; subsequently, covariates of interest were chosen in multivariable models via a stepwise selection approach. BUN, Blood Urea Nitrogen; RF, Respiratory Frequency; WBC, White blood cell count.

After adjustment, in the model for in-hospital mortality, CXCL10 emerged as a highly associated biomarker: for every 1,000 pg/mL increase, the risk of death increases by 26.0% (OR = 1.260; 95%CI: 1.173 - 1.353). Among clinical parameters, a 10 breaths/min increase in RF and a 10 mg/dL increase in BUN were associated respectively with 37.6% and 9.3% higher mortality risk, respectively (OR = 1.376; 95%CI: 1.195 - 1.584; OR = 1.093; 95%CI: 1.018 - 1.174). By contrast, the number of comorbidities acts as a protective effect on the outcome (OR = 0.955; 95%CI: 0.901- 0.994) (**Figure 3**).

In the model for ICU admission, CXCL10 and high RF again emerged as drivers of severity (OR = 1.133; 95%CI: 1.059 - 1.214; OR = 1.202; 95%CI: 1.048 - 1.379, respectively). Notably, TNF-α serum levels and number of comorbidities showed a trend toward significance in this model (OR = 1.239; 95%CI: 0.998 - 1.539; OR = 0.960; 95%CI: 0.916 - 1.006, respectively), suggesting a potential but weaker contribution among cytokines and clinical aspects (**Figure 3**).

For the composite severity outcome (death or ICU admission), the predictors included CXCL10 (OR = 1.214; 95%CI: 1.123 - 1.313), RF (OR = 1.489; 95%CI: 1.276 - 1.738), BUN (OR = 1.136; 95%CI: 1.050 - 1.228), and number of comorbidities (OR = 0.955; 95%CI: 0.904 - 1.008) (**Figure 3**).

## Discussion

In this retrospective study, sera from COVID-19 patients hospitalized during the first wave of the pandemic were analyzed to identify early immunological markers of disease severity. The integration of demographic and clinical data with serum cytokine and chemokine profiling enabled a comprehensive assessment of the role of circulating inflammation in shaping disease outcomes for the COVID-19 cases identified at that time. While viral sequencing was not performed for this cohort, the study timeframe (January–May 2020) aligns with the period when the SARS-CoV-2 ancestral strain was the predominant circulating lineage globally. Nonetheless, the absence of genomic confirmation remains a limitation of this study. Worth of note, the study cohort comprised SARS-CoV-2 naïve subjects, which allowed us to evaluate host immune profiles without confounding factors such as prior infection or vaccination. The most interesting result of our investigation is the identification of CXCL10 as one of the primary predictors of both ICU admission and in-hospital mortality. In our cohort, the prognostic value of this chemokine is significantly greater than that of cytokines more frequently associated with disease severity (21, 22, 36, 37). Through multivariable analysis, we delineated distinct but overlapping risk profiles for ICU admission and in-hospital mortality. ICU admission was driven by an acute inflammatory response, reflected by elevated CXCL10, TNF-α, and high respiratory rate. In contrast, fatal outcomes were associated with immune dysregulation sustained by CXCL10, together with organ dysfunction, as indicated by elevated BUN, and patient frailty. The composite outcome was driven by the same determinants as mortality.

A striking result of our study is the superior prognostic accuracy of serum CXCL10 compared with IL-6, which failed to reach statistical significance in our multivariable mortality model. While circulating IL-6 is a well-established driver of the acute-phase response and has been extensively studied in COVID-19 patients, its role in predicting fatal outcomes remains controversial: some studies report a significant positive correlation between serum IL-6 and mortality (20, 38) whereas others found only a non-statistically significant trend (39). A retrospective study further indicated that serum IL-6 alone may not accurately predict mortality once other covariates are considered (40). Similarly, a systematic review and meta-analysis concluded that while circulating IL-6 is an adequate predictor of severe disease, its isolated measurement is not associated with mortality in COVID-19 (41).

CXCL10 appears to act as a specific mechanistic driver of pulmonary damage rather than merely as a general inflammatory marker. While systemic IL-6 reflects overall inflammation, which is high in all hospitalized patients, CXCL10, via its receptor CXCR3, plays a central role in chemotaxis processes and elevated circulating levels would suggest more severe tissue injury (27, 42, 43). Moreover, interferon-induced secretion of CXCL10 drives the recruitment of activated Th1 lymphocytes, monocytes, and NK cells to sites of inflammation (44). Genetic association suggests that CXCL10 is implicated in susceptibility to pulmonary embolism in COVID-19 patients (45) and the correlation between plasma and epithelial lining fluid CXCL10 concentrations and the number of ventilator-free days, suggested a role in prolonged mechanical ventilation during ARDS in SARS-CoV-2 infection (46). Additionally, preclinical studies demonstrate that inhibition of CXCL10 and IFN-γ ameliorates myocardial injury following SARS-CoV-2 mRNA vaccination, highlighting its role in driving end-organ damage (47). Within this complex inflammatory network, our multivariable analysis highlights the potential role of serum TNF-α as a predictor of ICU admission. In our cohort, four of the five ICU patients who died had elevated TNF-α levels compared with survivors, although this difference did not reach statistical significance. Previous studies have suggested that systemic TNF-α inhibition may reduce mortality in COVID-19 patients (48). These findings indicate that TNF-α contributes to the cytokine storm, but acute hyperinflammation alone does not necessarily lead to fatal outcomes unless accompanied by additional factors such as advanced age and multi-organ dysfunction. Ultimately, persistent tissue injury driven or accompanied by CXCL10 appears to be the critical determinant of survival.

Our study showed that BUN and RF were associated with increased risk of severe outcome in COVID-19 patients. Although COVID-19 is primarily a respiratory infection, it is well recognized that the virus can also cause multi-organ involvement. In particular, renal impairment is common, as the angiotensin-converting enzyme 2 (ACE-2) receptor is expressed in multiple renal cell types (49). Our findings align with previous studies showing that elevated BUN is strongly associated with COVID-19 severity (50, 51). Similarly, elevated RF in our cohort correlate with the progression to acute respiratory failure, consistent with reports identifying RF as a biomarker for critical clinical deterioration (52, 53).

In our multivariate analysis, the presence of comorbidities appeared protective against ICU admission and death. This association should be interpreted as a consequence of the clinical approach rather than as a direct biological protective effect of comorbidities. Indeed, this apparently counterintuitive finding likely reflects the fact that patients with comorbidities received earlier and more intensive monitoring and intervention, which may have reduced their risk to progression to severe outcome.

Limitations of our study include its retrospective, single-center design, the relatively small sample size, and the limited number of severe clinical outcomes (ICU admissions and deaths), which may constrain the statistical power of the multivariable analysis. Cytokine measurements were performed only at hospital admission, which allowed us to identify CXCL10 as an early biomarker of severity, but precluded analysis of longitudinal kinetics and correlation with disease progression. Furthermore, as this study was conducted in early 2020, our findings reflect the host response to the original SARS-CoV-2 ancestral strain in an immunologically naive population; results may differ for subsequent variants of concern.

Overall, the present study identifies the chemokine CXCL10 as the strongest predictor of ICU admission and mortality in a cohort of naïve COVID-19 patients. From a translational perspective, integrating CXCL10 measurements into clinical assessments could be helpful in early risk stratification of hospitalized COVID-19 patients and to understand the probable outcome. Furthermore, CXCL10 could represent a promising therapeutic target, and its inhibition may provide an innovative strategy for the treatment of severe COVID-19.

## Data Availability

The raw data supporting the conclusions of this article will be made available by the authors, without undue reservation.

## Supplementary material

Supplementary Table S1 Characteristics of COVID-19 patients collected for the study analysis. The percentage of missing values is calculated on 103 units, the number of patients included in the population study. Data in parentheses represent percentages; the rates are presented to two decimal places.

Supplementary Table S2 P-values from univariable logistic regression for study outcomes, restricted to variables eligible for multivariable analysis in at least one outcome (p ≤ 0.200). The rates are presented to three decimal places and are calculated on 103 units, the number of patients included in the population study. For variables with a percentage of missing values not exceeding 15%, multiple imputation was performed via predictive mean matching (PMM), generating 5 imputed datasets. Final estimates were combined using Rubin’s rules to account for imputation uncertainty. Covariates showing an association, i.e. odds ratio (OR), with a p-value less than or equal to 0.20 in univariate analyses were considered eligible for inclusion in the corresponding multivariable models. (BUN, Blood Urea Nitrogen; CPAP, Continuous Positive Airway Pressure ventilation; CRP, C-reactive protein; DPR, D-dimer/Platelet Ratio; RF, Respiratory Frequency; LDH, Lactate Dehydrogenase; NLR, Neutrophil-to-Lymphocyte Ratio; PSI, Pneumonia Severity Index; WBC, White Blood Cell count.

Supplementary Table S3 Distribution of other characteristics of the study population not included in main text. Data in parentheses represent percentages or Inter-Quartile Range (IQR). The rates are presented to two decimal places and are calculated on 103 units, the number of patients included in the population study.
