# Supplementary material for: Serum cytokine profiling reveals CXCL10 (IP-10) as a major predictor of severe COVID-19 outcomes in hospitalized patients during the first pandemic wave in Italy
Source: Front Immunol. 2026 May 25;17:1816573. doi: 10.3389/fimmu.2026.1816573 (PMC13243234; doi:10.3389/fimmu.2026.1816573)
Supplement: Supplementary Table S1 — Characteristics of COVID-19 patients collected for the study analysis. The percentage of missing values is calculated on 103 units, the number of patients included in the population study. Data in parentheses represent percentages; the rates are presented to two decimal places. [file Table1.docx]

**Table S1** - Characteristics of COVID-19 patients collected for the study analysis

| **Characteristics** | **Units** | **Coding** | **Missing value**  **N (%)** |
| --- | --- | --- | --- |
| **Gender** |  | M/F | 0 (0.00%) |
| **Age** | years | Continuous | 0 (0.00%) |
| **Age 65+** |  | Yes/No | 0 (0.00%) |
| **Date of birth** |  | Date | 0 (0.00%) |
| **Respiratory Frequency (RF)** | breaths/min | Continuous | 10 (10.75%) |
| **Hypoxemia (PaO₂ < 60 mmHg and SpO₂ < 90%)** |  | Yes/No | 0 (0.00%) |
| **Charlson index** | score | Continuous | 2 (1.94%) |
| **History of autoimmune disease** |  | Yes/No | 7 (6.80%) |
| **CURB-65 score** | scale | 0-2 | 4 (3.88%) |
| **Extended CURB-65 score** | scale | 0-5 | 4 (3.88%) |
| **Pneumonia Severity Index (PSI)** |  | Continuous | 0 (0.00%) |
| **Date of hospital admission** |  | Date |  |
| **Hospital length of stay** | days | Continuous | 4 (4.04%) |
| **Oxygen therapy** |  | Yes/No | 14 (13.59%) |
| **Continuous Positive Airway Pressure (CPAP) ventilation** |  | Yes/No | 14 (13.59%) |
| **Neoplastic comorbidities** |  | Yes/No | 0 (0.00%) |
| **Cardiovascular comorbidities** |  | Yes/No | 0 (0.00%) |
| **Respiratory comorbidities** |  | Yes/No | 0 (0.00%) |
| **Renal comorbidities** |  | Yes/No | 0 (0.00%) |
| **Metabolic comorbidities** |  | Yes/No | 0 (0.00%) |
| **Neurological comorbidities** |  | Yes/No | 0 (0.00%) |
| **ICU admission** |  | Yes/No | 0 (0.00%) |
| **Death** |  | Yes/No | 0 (0.00%) |
| **White blood cell (WBC) count** | /µL | Continuous | 0 (0.00%) |
| **Neutrophil count** | /µL | Continuous | 0 (0.00%) |
| **Lymphocyte count** | /µL | Continuous | 0 (0.00%) |
| **Lymphocyte count > 1000**/µL |  | Yes/No | 0 (0.00%) |
| **Monocyte count** | /µL | Continuous | 0 (0.00%) |
| **C-reactive protein (CRP) > 41.8 mg/L** |  | Yes/No | 0 (0.00%) |
| **D-dimer** | ng/mL | Continuous | 9 (8.74%) |
| **Albumin** | g/L | Continuous | 8 (7.77%) |
| **Lactate Dehydrogenase (LDH)** | U/L | Continuous | 1 (0.97%) |
| **Platelet count** | /µL | Continuous | 0 (0.00%) |
| **Platelet count > 150000/µL** |  | Yes/No | 0 (0.00%) |
| **Blood Urea Nitrogen (BUN)** | mg/dL | Continuous | 1 (0.97%) |
| **Glucose** | mg/dL | Continuous | 3 (2.91%) |
| **Sodium** | mmol/L | Continuous | 8 (7.77%) |
| **CXCL10** | Pg/mL | Continuous | 0 (0.00%) |
| **IL-6** | Pg/mL | Continuous | 1 (0.97%) |
| **IL-8** | Pg/mL | Continuous | 0 (0.00%) |
| **CCL3** | Pg/mL | Continuous | 0 (0.00%) |
| **TNF-α** | Pg/mL | Continuous | 1 (0.97%) |
| **IL-1β** | Pg/mL | Continuous | 0 (0.00%) |
| **IL-10** | Pg/mL | Continuous | 0 (0.00%) |
| **Call score** | score | Continuous | 0 (0.00%) |
| **Plaquenil** |  | Yes/No | 2 (1.94%) |
| **Antiviral therapy** |  | Yes/No | 5 (4.85%) |
| **Tocilizumab** |  | Yes/No | 7 (6.80%) |
| **Azithromycin** |  | Yes/No | 5 (4.85%) |
| **Probiotic therapy** |  | Yes/No | 5 (4.85%) |
| **CD38 concentration** | pg/mL | Continuous | 1 (0.97%) |

*The percentage of missing values is calculated on 103 units, the number of patients included in the population study. Data in parentheses represent percentages; the rates are presented to two decimal places.*
