# Supplementary material for: Serum cytokine profiling reveals CXCL10 (IP-10) as a major predictor of severe COVID-19 outcomes in hospitalized patients during the first pandemic wave in Italy
Source: Front Immunol. 2026 May 25;17:1816573. doi: 10.3389/fimmu.2026.1816573 (PMC13243234; doi:10.3389/fimmu.2026.1816573)
Supplement: Supplementary Table S3 — Distribution of other characteristics of the study population not included in main text. Data in parentheses represent percentages or Inter-Quartile Range (IQR). The rates are presented to two decimal places and are calculated on 103 units, the number of patients included in the population study. [file Table2.docx]

**Table S2** - Distribution of other characteristics of the study population not included in main text

| **Characteristics** | | **Cases**  **N (%)** |
| --- | --- | --- |
| **Call score (Median [IQR])** | | 9 [7-11] |
| **Plaquenil** | Yes | 94 (91.26%) |
|  | No | 7 (6.80%) |
| **Antiviral therapy** | Yes | 38 (36.89%) |
|  | No | 60 (58.25%) |
| **Tocilizumab** | Yes | 50 (48.54%) |
|  | No | 46 (44.66%) |
| **Zitromax** | Yes | 33 (32.04%) |
|  | No | 65 (63.11%) |
| **Probiotic therapy** | Yes | 44 (42.72%) |
|  | No | 54 (52.43%) |
| **CD38 concentration (Median [IQR])** | | 0 [0 - 1] |

*Data in parentheses represent percentages or Inter-Quartile Range (IQR). The rates are presented to two decimal places and are calculated on 103 units, the number of patients included in the population study.*
